# Real-world outcomes of cefepime/enmetazobactam for Enterobacterales infections: a retrospective descriptive study

**DOI:** 10.1128/spectrum.02002-26

**Published:** 2026-08-07

**Authors:** Cecilia Bonazzetti, Marco Falcone, Giusy Tiseo, Francesco Di Gennaro, Luisa Frallonardo, Annalisa Saracino, Sara Tedeschi, Sergio Lo Caputo, Irene Francesca Bottalico, Filippo Del Puente, Emanuele Pontali, Alessandro Cilli, Linda Bussini, Michele Bartoletti, Alessandro Russo, Luca Pipito, Antonio Cascio, Ilaria De Benedetto, Silvia Corcione, Francesco Giuseppe De Rosa, Alberto Farese, Bruno Viaggi, Alessandra Oliva, Claudio Mastroianni, Martina Ranzenigo, Antonella Castagna, Fabrizio Pulvirenti, Luca Guerra, Maddalena Giannella, Pierluigi Viale

**Affiliations:** 1 Infectious Diseases Unit, IRCCS Azienda Ospedaliero-Universitaria di Bologna 18508, Bologna, Italy; 2 Department of Medical and Surgical Sciences, University of Bologna 9296, Bologna, Italy; 3 Infectious Diseases Unit, Department of Clinical and Experimental Medicine, Azienda Ospedaliera Universitaria Pisana, University of Pisa 9310, Pisa, Italy; 4 Department of Precision and Regenerative Medicine and Ionian Area (DiMePRe-J), Clinic of Infectious Diseases, University of Bari "Aldo Moro" 9295, Bari, Italy; 5 Clinic of Infectious Diseases, Department of Medical and Surgical Sciences, University of Foggia 18972, Foggia, Italy; 6 Department of Infectious Diseases, Ente Ospedaliero Ospedali Galliera 9336, Genoa, Italy; 7 Infectious Disease Unit, IRCCS Humanitas Research Hospital 9268, Milan, Italy; 8 Department of Biomedical Sciences, Humanitas University, Milan, Italy; 9 Department of Medical and Surgical Sciences, "Magna Graecia" University 335566, Catanzaro, Italy; 10 Department of Health Promotion, Mother and Child Care, Internal Medicine and Medical Specialties "G D'Alessandro", University of Palermo 18998, Palermo, Italy; 11 Department of Medical Sciences, Infectious Diseases, University of Turin 9314, Turin, Italy; 12 Infectious Diseases Clinic, Careggi University Hospital, Firenze, Italy; 13 Intensive Care Unit CTO, Careggi University Hospital, Firenze, Italy; 14 Department of Public Health and Infectious Diseases, Sapienza Università di Roma 9311 , Rome, Italy; 15 Department of Internal Medicine, Endocrine-Metabolic Sciences and Infectious Diseases, AOU Policlinico Umberto I, Rome, Italy; 16 Unit of Infectious Diseases, IRCCS San Raffaele Scientific Institute 9372, Milan, Italy; 17 Unit of Infectious Diseases, Gela Hospital, Gela, Italy; 18 Infectious Diseases Unit, Department for Integrated Infectious Risk Management, IRCCS Policlinico S. Orsola-Malpighi Hospital, AUSL Bologna-IRCCS IOR Bologna-AUSL, Imola, Italy; Tel Aviv Sourasky Medical Center, Tel Aviv, Israel

**Keywords:** gram-negative, extended-spectrum beta-lactamase, cefepime/enmetazobactam

## Abstract

Cefepime/enmetazobactam (FEP/META) is a novel β-lactam/β-lactamase inhibitor combination with activity against Enterobacterales, including extended-spectrum β-lactamase-producing isolates, but real-world clinical data outside randomized trials remain limited. We conducted a retrospective multicenter descriptive study across 13 Italian hospitals. Eligible patients had infections caused by Enterobacterales and received ≥72 h of cefepime/enmetazobactam therapy (alone or in combination with other antimicrobials) under a compassionate-use program. Thirty-two patients were included. The most frequently isolated pathogens were *Escherichia coli* (16/32, 50.0%) and *Klebsiella pneumoniae* (13/32, 40.6%). Extended-spectrum β-lactamase production was documented in 29/32 isolates (90.6%), while β-lactam/β-lactamase inhibitor resistance and fluoroquinolone resistance were each observed in 18/32 isolates (56.2%). Four isolates were ertapenem-resistant but remained susceptible to imipenem and meropenem; no carbapenemase production was confirmed. FEP/META was safely administered as targeted therapy in most cases and was used both as monotherapy and in combination regimens, according to clinical severity and source of infection. Clinical cure was achieved in all patients, and microbiological eradication was documented in 25/32 cases (78.1%). No in-hospital deaths were recorded. Despite the inherent limitations of retrospective real-world studies, our findings suggest that cefepime/enmetazobactam may be a well-tolerated component of carbapenem-sparing treatment strategies for selected documented Enterobacterales infections. In 32 real-world documented Enterobacterales infections, cefepime/enmetazobactam achieved 100% clinical cure and 78.1% microbiological eradication, with favorable tolerability.

**IMPORTANCE:**

Antibiotic-resistant infections are an increasing challenge, and clinicians need effective treatments that can reduce reliance on carbapenems, which are often considered last-line antibiotics. Cefepime/enmetazobactam is a new antibiotic combination with activity against resistant Enterobacterales, particularly ESBL-producing bacteria, but real-world clinical data remain limited. This multicenter Italian study describes its use in 32 patients with Enterobacterales infections, including urinary tract, bloodstream, and respiratory infections. Clinical cure was achieved in all patients, microbiological eradication was documented in most cases, and no in-hospital deaths occurred. These findings suggest that cefepime/enmetazobactam may be a useful and well-tolerated carbapenem-sparing option for selected resistant gram-negative infections, while emphasizing the need for larger studies to confirm its role in routine clinical practice.

## INTRODUCTION

Extended-spectrum β-lactamase (ESBL)-producing Enterobacterales are a major cause of clinically significant infections and remain a substantial therapeutic challenge because they limit the activity of many standard β-lactams and often drive carbapenem use (1). Cefepime/enmetazobactam (FEP/META) combines cefepime with enmetazobactam, a novel β-lactamase inhibitor that restores cefepime activity against ESBL-producing Enterobacterales and selected serine β-lactamases (2). *In vitro*, FEP/META has shown potent activity against many ESBL-producing Enterobacterales, whereas activity is less consistent against carbapenemase-producing isolates, particularly metallo-β-lactamase producers, and remains less well defined against non-fermenting gram-negative bacilli (3–6). Real-life clinical evidence for FEP/META is still limited. Its development has been supported primarily by a phase 3 randomized trial in patients with complicated urinary tract infection (cUTI) or acute pyelonephritis, in which FEP/META achieved favorable clinical and microbiological outcomes compared with piperacillin/tazobactam (2). However, beyond the registration setting, available data are still derived largely from microbiological studies, pharmacokinetic/pharmacodynamic investigations, and narrative reviews rather than from real-world clinical studies (3–7). We therefore conducted a retrospective multicenter descriptive study to evaluate the real-world outcomes of cefepime/enmetazobactam for documented Enterobacterales infections treated in routine clinical practice.

## MATERIALS AND METHODS

We conducted a retrospective, multicenter descriptive study across 13 Italian hospitals to describe real-world outcomes of FEP/META used under a compassionate-use program for documented Enterobacterales infections treated in routine clinical practice. Consecutive adult patients treated between January and December 2025 were screened. Eligible patients had a microbiologically documented Enterobacterales infection and received FEP/META for at least 72 h, either as monotherapy or in combination with other antimicrobials (the combined therapy was not used as rescue therapy for clinical deterioration or lack of response to FEP/META); patients treated for <72 h or without microbiological documentation were not considered evaluable. The 72-h threshold was defined *a priori* to ensure sufficient exposure to FEP/META for assessment of clinical response and tolerability and avoid attributing outcomes to very brief or interrupted treatment courses. During the study period, FEP/META was not routinely available in the participating hospitals and was accessed through a compassionate-use program. Approval for its use was obtained by the treating physicians from each local ethics committee on a case-by-case basis, in accordance with national regulations for expanded access to investigational drugs. The decision to request FEP/META was based on clinical judgment and microbiological findings and was mainly driven by the need for a targeted carbapenem-sparing option in patients with documented Enterobacterales infections, particularly ESBL-producing isolates, prior healthcare and antibiotic exposure, or limited non-carbapenem treatment options.

Microbiological identification and antimicrobial susceptibility testing were performed at each participating center according to local laboratory procedures. Resistance mechanisms, including ESBL production and carbapenem resistance, were recorded when reported by local microbiology laboratories. β-lactam/β-lactamase inhibitor resistance was defined as resistance or non-susceptibility to piperacillin/tazobactam and/or amoxicillin/clavulanate. Ertapenem-only-resistant Enterobacterales were defined as isolates resistant to ertapenem but susceptible to imipenem and meropenem, according to local antimicrobial susceptibility testing reports. Systematic molecular characterization of resistance determinants was not performed, and isolates were not centrally stored or re-tested. Repeat FEP/META MIC testing was not routinely available.

Clinical and microbiological data were retrospectively collected from medical records using a standardized case report form. Variables included age, sex, Charlson comorbidity index, ward submitting the index culture, infection source, acquisition setting, SOFA score at infection onset, microbiological findings, timing of FEP/META initiation, treatment duration, combination therapy, clinical outcomes, and prior hospitalization or antibiotic exposure within 3 months before the index infection. Clinical severity at FEP/META initiation was not systematically captured in the case report form. Antimicrobial therapy administered before FEP/META initiation was not systematically captured in the case report form, except when specifically reported by the participating centers. Infections were classified as complicated urinary tract infection, bloodstream infection, lower respiratory tract infection, or other infection. Complicated urinary tract infection was defined as the presence of urinary tract signs or symptoms and/or systemic signs of infection, together with a positive urine culture yielding the baseline Enterobacterales isolate and at least one complicating factor, including structural or functional urinary tract abnormality, urinary catheter, nephrostomy, renal stone, urinary obstruction, renal transplantation, or relevant comorbidity. Bloodstream infection was defined as isolation of an Enterobacterales species from at least one blood culture in the presence of compatible clinical signs of infection. Lower respiratory tract infection was defined as compatible respiratory signs or symptoms, radiological evidence of pulmonary infection, and microbiological documentation from respiratory samples or blood cultures. Other infections were classified according to the primary site of infection when supported by compatible clinical and/or radiological findings and microbiological documentation. Infection onset was categorized as hospital- or community-acquired according to symptom onset. Sepsis and septic shock were defined according to contemporary international criteria (8). Clinical cure was assessed at the end of FEP/META therapy and was defined as resolution or substantial improvement of infection-attributable signs and symptoms, without need for escalation, discontinuation, or switch to another antimicrobial regimen because of lack of clinical response. Infection-attributable signs and symptoms included fever, hemodynamic instability, leukocytosis or elevated inflammatory markers, source-specific symptoms, and organ dysfunction when considered attributable to infection. Extension of FEP/META duration because of slow but progressive clinical improvement was not considered clinical failure if resolution was ultimately achieved without antimicrobial escalation. Conversely, discontinuation, escalation, or switch due to clinical deterioration or lack of response was considered clinical failure. Planned combination therapy at FEP/META initiation was not considered clinical failure. Moreover, addition of another antimicrobial agent after FEP/META initiation because of clinical deterioration or lack of response was considered treatment escalation and classified as clinical failure.

Microbiological eradication was defined as documented absence of the baseline pathogen in follow-up cultures obtained from blood and/or the primary infection site after FEP/META initiation. Follow-up cultures were performed according to clinical judgment and local practice and were not mandated by protocol. Patients without available follow-up cultures were classified as not assessable for microbiological eradication and were not assumed to have achieved microbiological success. Recurrence was defined as a new infectious episode caused by the same pathogen within 30 days after treatment completion. In-hospital mortality was defined as death from any cause during the index hospitalization. Adverse events potentially related to FEP/META were specifically collected through the standardized case report form. Investigators were asked to review medical records, clinical notes, and laboratory data and report adverse events occurring during FEP/META therapy that were considered potentially drug-related by the treating physicians. Collected events included neurological events, gastrointestinal events, including *Clostridioides difficile* infection, hypersensitivity reactions, renal or hepatic laboratory abnormalities, electrolyte disturbances, and treatment discontinuation due to toxicity. The primary outcome was clinical cure. Secondary outcomes were microbiological eradication, recurrence, in-hospital mortality, and adverse events occurring during FEP/META therapy and considered potentially related to treatment by the treating physicians. Analyses were descriptive: categorical variables were reported as number and percentage, and continuous variables as median (IQR) or mean (SD), as appropriate. Given the small sample size and retrospective descriptive study design, no formal comparative analyses were performed. A descriptive subgroup analysis was performed according to infection type, comparing patients with complicated urinary tract infection with those with non-cUTI infections, including bloodstream infection, lower respiratory tract infection, and other infection sources. Statistical analyses were conducted using IBM SPSS Statistics.

## RESULTS

### Clinical and microbiological characteristics

**Fig 1:**
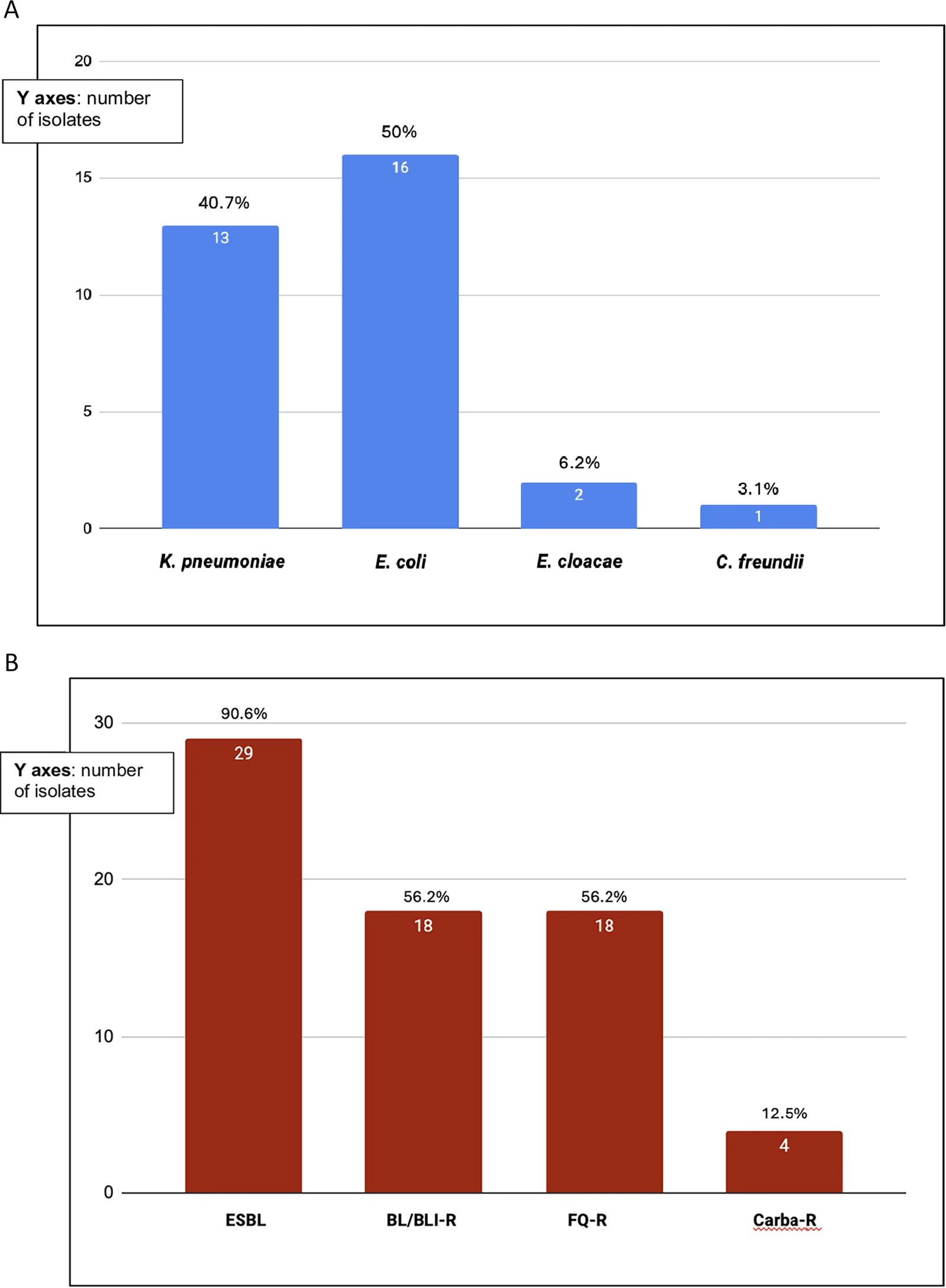
Pathogen distribution and resistance phenotypes. (**A**) Distribution of Enterobacterales species isolated in the study sample. (**B**) Distribution of resistance phenotypes among baseline isolates. ESBL, extended-spectrum β-lactamase; BL/BLI, β-lactam/β-lactamase inhibitor resistance, defined as resistance or non-susceptibility to piperacillin/tazobactam and/or amoxicillin/clavulanate; FQ, fluoroquinolone; and Carba-R, carbapenem resistance.

Overall, 32 infections were included in the study (Table 1). Most patients were male (22/32, 68.7%), with a median age of 69 years (IQR, 56–78), and had a substantial burden of comorbidity, with a median Charlson comorbidity index of 5 (IQR, 3–8). Both previous hospitalization and antibiotic exposure were documented in 26 patients (81.2%). The median SOFA score at infection onset was 2 (IQR, 1–3). Twenty infections (62.5%) were hospital-acquired, whereas 12 (37.5%) were community-acquired. Most index cultures were obtained in medical wards (26/32, 81.2%), while three cases each (9.4%) originated from surgical wards and intensive care units. Complicated urinary tract infection was the most frequent source (18/32, 56.2%), followed by bloodstream infection (8/32, 25.0%), lower respiratory tract infection (4/32, 12.5%), and other infections (2/32, 6.2%). For subgroup analysis, patients were stratified into cUTI (18/32, 56.2%) and non-cUTI infections (14/32, 43.8%). The non-cUTI group included bloodstream infections (8/14, 57.1%), lower respiratory tract infections (4/14, 28.6%), and other infection sources (2/14, 14.3%). Baseline characteristics according to infection type are shown in Table 1. *Escherichia coli* was the most frequently isolated pathogen (16/32, 50%), followed by *Klebsiella pneumoniae* (13/32, 40.6%), *Enterobacter cloacae* (2/32, 6.2%), and *Citrobacter freundii* (1/32, 3.1%) (Fig. 1).

**TABLE 1:** Characteristics of the study sample according to infection type^*a*^^,^^*b*^

Patient characteristics	*n* = 32	cUTI *n* = 18	non-cUTI *n* = 14
Age (years), median (IQR)	69 (56–78)	70.5 (60–79)	67.5 (53–78)
Gender (male, %)	22 (68.7%)	13 (72.2%)	9 (64.3%)
CCI, median (IQR)	5 (3–8)	5.5 (4–10)	3.5 (2–7)
Pre-infection healthcare interventions			
Previous hospitalization and antibiotic therapy exposure	26 (81.2%)	15 (83.3%)	11 (78.6%)
Infection characteristics			
Hospital acquired	20 (62.5%)	11 (61.1%)	9 (64.3%)
Community acquired	12 (37.5%)	7 (38.9%)	5 (35.7%)
Source of infection			
cUTI	18 (56.2%)	18 (100%)	–
BSI	8 (25%)	–	8 (57.1%)
LRTI	4 (12.5%)	–	4 (28.6%)
Other	2 (6.2%)	–	2 (14.3%)
SOFA score at infection onset	2 (1–3)	2 (1–2)	3 (2–4)
Ward submitting index culture			
Medical	26 (81.2%)	15 (83.3%)	11 (78.6%)
Surgical	3 (9.4%)	1 (5.6%)	2 (14.3%)
ICU	3 (9.4%)	2 (11.1%)	1 (7.1%)

^*a*^ CCI, Charlson comorbidity index; cUTI, complicated urinary tract infection; BSI, bloodstream infection; LRTI, lower respiratory tract infection; and ICU, intensive care unit.

^*b*^ An en dash indicates that no patients were present in that category within the corresponding subgroup.

Resistance phenotypes were dominated by ESBL production, which was documented in 29/32 isolates (90.6%). Resistance or non-susceptibility to β-lactam/β-lactamase inhibitor combinations and fluoroquinolone resistance were each observed in 18/32 isolates (56.2%), whereas carbapenem resistance was less frequent (4/32, 12.5%). All four carbapenem-resistant isolates were ertapenem-resistant but remained susceptible to imipenem and meropenem, consistent with an ertapenem-only-resistant Enterobacterales phenotype. No carbapenemase production was confirmed by local microbiology reports, and no isolates were reported as producing OXA-48, *Klebsiella pneumoniae* carbapenemase (KPC), or metallo-β-lactamases. Patient-level details of these four cases are provided in Table S1. FEP/META MIC testing was available for nine isolates (28.1% of 32 evaluable strains). Recorded MIC values were generally low, ranging from 0.032 to 1.5 mg/L (median, 0.06 mg/L), with most values clustering below 0.25 mg/L and only three isolates showing MICs of 1–1.5 mg/L.

### Treatment and outcomes

All 32 patients included in the analysis received FEP/META for at least 72 h. Treatment duration ranged from 3 to 28 days, with a median treatment duration of 9 days (IQR, 5–10). Cefepime/enmetazobactam was initiated after a median of 4 days (IQR, 3–7) from infection onset, and it was administered as monotherapy in most cases (29/32, 90.6%), whereas three patients (9.4%) received combination therapy, all with fosfomycin (Table 2). In all three patients receiving combination therapy, fosfomycin was started concomitantly with FEP/META as part of the initial FEP/META-containing regimen. Nine patients (28.1%) started FEP/META ≥7 days after infection onset. Previous meropenem therapy was explicitly reported in one patient who started FEP/META 13 days after infection onset. Dose adjustment for renal function was required in 10 patients (31.2%). Clinical cure was achieved in all 32 patients (100%). Documented microbiological eradication was observed in 25/32 patients (78.1%). The remaining seven patients (21.9%) had no documented microbiological eradication assessment and were classified as not assessable rather than as microbiological failures. When stratified by infection type, documented microbiological eradication occurred in 13/18 patients with cUTI (72.2%) and in 12/14 patients with non-cUTI infections (85.7%). No patient had documented persistence of the baseline pathogen in follow-up cultures. Only two patients (6.2%) experienced an in-hospital recurrence of infection after treatment discontinuation; in one case, recurrence was likely attributable to an infected kidney stone that had not been eradicated. No in-hospital deaths were observed.

**TABLE 2:** Treatments and outcomes^*a*^

FEP/META treatment variables	*N* = 32	cUTI *n* = 18	non-cUTI *n* = 14
Days before FEP/META treatment, median (IQR)	4 (3–7)	5 (3–7)	4 (3–7)
Monotherapy regimens	29 (90.6%)	18 (100%)	11 (78.6%)
Combination regimens	3 (9.4%)	–	3 (21.4%)
Fosfomycin	3 (9.4%)	–	3 (21.4%)
Days of treatment, median (IQR)	9 (5–10)	6 (5–10)	10 (8–12)
Dose adjusted for renal function	10 (31.2%)	6 (33.3%)	4 (28.6%)
Outcomes			
Clinical cure	32 (100%)	18 (100%)	14 (100%)
Microbiological eradication	25 (78.1%)	13 (72.2%)	12 (85.7%)
In-hospital infection recurrence	2 (6.2%)	1 (5.6%)	1 (7.1%)
Adverse events	3 (9.4%)	2 (11.1%)	1 (7.1%)
In-hospital mortality	0 (0%)	0 (0%)	0 (0%)

^*a*^ An en dash indicates that no patients were present in that category within the corresponding subgroup.

### Adverse events

Cefepime/enmetazobactam was generally well tolerated (Table 2). Three adverse events (9.4%) were reported during treatment, including *Clostridioides difficile* colitis, hyponatremia, and neurotoxicity. The latter two events resolved promptly after treatment discontinuation, and no fatal adverse events occurred.

## DISCUSSION

In this multicenter real-world descriptive study, cefepime/enmetazobactam was associated with high clinical cure and favorable microbiological outcomes in documented Enterobacterales infections, mostly due to ESBL-producing isolates.

Microbiological eradication was lower than clinical cure in our study sample and was slightly lower, in percentage terms, than that reported in the cefepime/enmetazobactam arm of the registrational trial (78.1% vs 82.9%) (2). However, this comparison should be interpreted cautiously because of differences in study design, enrolled populations, infection syndromes, and microbiological follow-up procedures. In routine clinical practice, follow-up cultures are less standardized than in clinical trials, and microbiological endpoints may therefore underestimate treatment success (3, 9). Stratification by infection type is clinically relevant because most randomized evidence for FEP/META derives from patients with complicated urinary tract infection, whereas data in bloodstream infections, respiratory tract infections, and other non-cUTI syndromes remain limited. In our study sample, 14 patients had non-cUTI infections, including bloodstream infections, lower respiratory tract infections, and other infection sources. Although favorable clinical and microbiological outcomes were also observed in this subgroup, with documented microbiological eradication in 12/14 patients (85.7%), these findings should be interpreted with caution because of the small sample size, clinical heterogeneity, lack of comparator group, and incomplete standardized microbiological follow-up. Therefore, our findings in non-cUTI infections should be considered exploratory and hypothesis-generating, and dedicated prospective studies are needed to define the role of FEP/META in these infection syndromes.

A key implication is its potential as a carbapenem-sparing strategy. Preserving carbapenems remains an antimicrobial stewardship priority, especially in settings with high ESBL prevalence and risk of selecting more resistant phenotypes. In this context, the *in vitro* profile of FEP/META and the favorable outcomes observed here support its use as a targeted carbapenem-sparing option for selected ESBL-producing Enterobacterales infections, when susceptibility is documented, and clinical conditions are appropriate (3, 9, 10). However, this interpretation is mainly applicable to the species distribution observed in our study population, which was dominated by *E. coli* and *K. pneumoniae*. Species with inducible chromosomal AmpC production, particularly *Enterobacter* spp., were underrepresented. This is relevant because FEP/META MIC50 and MIC90 values may be higher among AmpC-producing Enterobacterales than among *E. coli* and *Klebsiella* spp., limiting the generalizability of our findings to this microbiological subgroup (10).

From a mechanistic perspective, enmetazobactam is a penicillanic acid sulfone β-lactamase inhibitor, structurally related to tazobactam but distinct from several newer β-lactamase inhibitor classes, including diazabicyclooctane- and boronate-based inhibitors (3). This distinction may be clinically relevant because resistance mechanisms emerging under enmetazobactam exposure may not necessarily confer cross-resistance to other β-lactam/β-lactamase inhibitor combinations used for carbapenemase-producing organisms. Nevertheless, clinical data on the emergence of enmetazobactam resistance and its relationship with susceptibility to other β-lactamase inhibitor combinations remain limited and warrant further investigation (3).

The role of FEP/META against carbapenem-resistant isolates remains less defined. In our study sample, four isolates were resistant to ertapenem but remained susceptible to imipenem and meropenem, consistent with an ertapenem-only-resistant Enterobacterales phenotype, and no carbapenemase production was confirmed. This phenotype may reflect non-carbapenemase mechanisms associated with ertapenem non-susceptibility, such as ESBL production combined with permeability changes, although molecular confirmation was not available. Therefore, these findings should not be extrapolated to carbapenemase-producing Enterobacterales, particularly KPC-, OXA-48-like-, or metallo-β-lactamase-producing isolates (6, 11). Although *in vitro* data suggest possible activity against some OXA-48-like producers, current evidence does not support a reliable role against KPC-producing isolates, and our study provides no direct evidence for confirmed carbapenemase-producing Enterobacterales (11, 12). The role of FEP/META outside Enterobacterales is even more uncertain: *in vitro* activity against *Pseudomonas aeruginosa* and, to a lesser extent, *Acinetobacter baumannii* has been reported, but evidence remains heterogeneous and insufficient for routine use (4, 5). Our study sample adds no information here because no non-fermenters were included.

From a practical perspective, FEP/META may also support outpatient parenteral therapy or early-discharge strategies, given its favorable stability in elastomeric devices, with possible benefits in hospitalization burden and costs (13, 14).

Cost considerations are also relevant to the real-world implementation of FEP/META. As with other recently introduced antimicrobial agents, acquisition cost may limit widespread use, particularly in healthcare settings with constrained antimicrobial budgets or restrictive reimbursement pathways. However, the overall economic impact of FEP/META cannot be inferred from drug acquisition cost alone and may depend on several factors, including local ESBL prevalence, carbapenem-sparing stewardship priorities, treatment duration, relapse prevention, length of hospital stay, and the feasibility of outpatient parenteral antimicrobial therapy or early discharge strategies (15). Our study was not designed to assess cost-effectiveness or budget impact, and dedicated pharmacoeconomic analyses are needed to define the value of FEP/META across different infection syndromes and healthcare systems (15).

More broadly, comparative studies vs meropenem are needed to define whether FEP/META can reliably serve as a carbapenem-sparing option across different infection syndromes and patient populations, particularly in immunocompromised and hematologic patients. Pediatric data are also lacking, although a phase 2 trial in children and adolescents with cUTI is ongoing (16).

This study has important limitations: its retrospective descriptive design, small sample size, lack of comparator group, incomplete microbiological characterization and follow-up cultures, predominance of *E. coli* and *K. pneumoniae*, underrepresentation of species commonly associated with inducible chromosomal AmpC production, absence of confirmed carbapenemase-producing isolates, overrepresentation of cUTI relative to non-cUTI infection syndromes, and absence of non-fermenters, all of which limit causal inference and generalizability. In addition, severity at the time of FEP/META initiation and individual infection-attributable signs and symptoms were not systematically captured in the case report form. Therefore, although clinical cure was assessed at the end of FEP/META therapy, we could not fully describe the trajectory of clinical improvement between infection onset and FEP/META initiation. This is particularly relevant because FEP/META was started after a median of 4 days from infection onset, and nine patients started treatment ≥7 days after infection onset. Moreover, microbiological outcomes should also be interpreted cautiously because follow-up cultures were not systematically obtained and were performed according to clinical judgment and local practice. Patients without follow-up cultures were not counted as microbiological successes, but the lack of standardized microbiological follow-up limited the assessment of microbiological eradication and may have led to underestimation or misclassification of microbiological outcomes. Another important limitation is that antimicrobial therapy administered before FEP/META initiation, including potential carbapenem exposure, was not systematically captured. Therefore, clinical cure cannot be attributed exclusively to FEP/META in all cases, particularly in patients who may have received active therapy before switching to FEP/META or who started FEP/META several days after infection onset. Our findings should consequently be interpreted as real-world outcomes of therapeutic strategies, including FEP/META, rather than as evidence of the independent efficacy of FEP/META in the absence of prior active therapy. Moreover, only three isolates belonged to species with potential inducible chromosomal AmpC production, including two *Enterobacter cloacae* isolates and one *Citrobacter freundii* isolate. Therefore, our findings cannot be confidently generalized to infections caused by AmpC-producing Enterobacterales, particularly *Enterobacter* spp., for which FEP/META MIC distributions may be less favorable. Another key limitation is the absence of confirmed carbapenemase-producing isolates. Although four isolates were ertapenem-resistant, all remained susceptible to imipenem and meropenem, and no carbapenemase production was confirmed. Therefore, our findings do not provide evidence for the use of FEP/META against confirmed carbapenemase-producing Enterobacterales, including KPC-, OXA-48-like-, or metallo-β-lactamase-producing isolates. Finally, adverse events were collected retrospectively from medical records through a standardized case report form, without prospective active surveillance or centralized causality adjudication. Therefore, underreporting or misclassification of adverse events cannot be excluded. Repeat susceptibility testing and MIC assessment of recurrent isolates were not systematically available. Therefore, we could not evaluate whether recurrence was associated with the emergence of reduced susceptibility to FEP/META or to other antimicrobial agents. Future prospective studies should incorporate centralized susceptibility testing, molecular characterization of baseline and follow-up isolates, and assessment of changes in FEP/META MIC values, particularly in patients with microbiological persistence or recurrence.

### Conclusions

In conclusion, FEP/META appeared to be a well-tolerated component of carbapenem-sparing treatment strategies for selected Enterobacterales infections, predominantly caused by ESBL-producing *E. coli* and *K. pneumoniae*, in this real-world multicenter descriptive study. Given the retrospective descriptive design, lack of comparator group, predominance of cUTI, underrepresentation of AmpC-associated species, absence of confirmed carbapenemase-producing isolates, incomplete microbiological characterization, and limited information on antimicrobial therapy before FEP/META initiation, larger prospective studies are needed to define its independent effectiveness across different infection syndromes, resistance mechanisms, and patient populations.

## Data Availability

De-identified individual-level data supporting the findings of this study are available from the corresponding author upon reasonable request, in accordance with applicable ethical, privacy, and institutional regulations. No genome sequence data, high-throughput data sets, or publicly deposited data sets were generated or analyzed in this study.

## SUPPLEMENTAL MATERIAL

Table S1 spectrum.02002-26-s0001.docx Characteristics of patients with ertapenem-only-resistant Enterobacterales (EORE) infections.

ASM does not own the copyrights to Supplemental Material that may be linked to, or accessed through, an article. The authors have granted ASM a non-exclusive, world-wide license to publish the Supplemental Material files. Please contact the corresponding author directly for reuse.
