# Supplementary material for: Real-world outcomes of cefepime/enmetazobactam for Enterobacterales infections: a retrospective descriptive study
Source: Microbiol Spectr. 2026 Aug 7;14(9):e02002-26. doi: 10.1128/spectrum.02002-26 (PMC13532315; doi:10.1128/spectrum.02002-26)
Supplement: Supplementary file 1 [file spectrum.02002-26-s0001.docx]

**Supplementary Table 1. Characteristics of patients with ertapenem-only-resistant Enterobacterales (EORE) infections.**

|  | **Patient 1** | **Patient 2** | **Patient 3** | **Patient 4** |
| --- | --- | --- | --- | --- |
| **Infection source** | cUTI | BSI | cUTI | cUTI |
| **SOFA score at infection onset** | 2 | 3 | 2 | 3 |
| **Ward submitting index culture** | Medical | Medical | Medical | Medical |
| **Pathogen** | *Escherichia coli* | *Escherichia coli* | *Klebsiella pneumoniae* | *Klebsiella pneumoniae* |
| **BL/BLI resistance** | No | No | Yes | Yes |
| **Ertapenem** | Resistant | Resistant | Resistant | Resistant |
| **Imipenem and Meropenem** | Susceptible | Susceptible | Susceptible | Susceptible |
| **Carbapenemase production confirmed** | No | No | No | No |
| **FEP/META treatment duration, days** | 7 | 10 | 7 | 3 |
| **Combination therapy** | No | No | No | No |
| **Clinical cure** | Yes | Yes | Yes | Yes |
| **Microbiological eradication** | Yes | Yes | Yes | Yes |
| **Recurrence** | No | No | No | No |

**Table note:** Ertapenem-only-resistant Enterobacterales were defined as isolates resistant to ertapenem but susceptible to imipenem and meropenem. Carbapenemase production was not confirmed in any of the four isolates. BL/BLI resistance was defined as resistance or non-susceptibility to piperacillin/tazobactam and/or amoxicillin/clavulanate. BSI, bloodstream infection; cUTI, complicated urinary tract infection; ESBL, extended-spectrum β-lactamase; FEP/META, cefepime/enmetazobactam.
